# Educational—the Dental Colleges

**Published:** 1890-09

**Authors:** 


### Educational—The Dental Colleges.

The time is near at hand when the regular work will begin in all the dental colleges in the country with one or two exceptions; and this year will perhaps witness a larger attendance at these institutions than ever before, though the number at the last term was larger than ever before—there were in actual attendance over twenty-seven hundred students—there were twenty-six hundred and seventy-three in twenty-seven colleges, and a few not reported.

This large number last year was due, in part at least, to the fact that after the present year three terms will be required for graduation by all reputable colleges, and the same influence doubtless will be the occasion of an increase influx at the coming term.

This state of affairs will throw a greater responsibility upon the colleges, or rather upon their faculties. Unquestionably many of those who will come forward for entrance will be wholly unqualified, in both natural and acquired ability, for taking up the required course of study and work, and if the proper course is pursued all such will be refused entrance. To do just what ought to be done under such circumstances is not always an easy task; it requires close discrimination, experience, and a good judge of human nature, and even with all these in exercise, mistakes will be made.

Another difficulty encountered, with a large number of students, is the greater difficulty in teaching, and especially is this experienced in the technical departments, in which proper instruction can only be given by frequent personal contact, and direction; it is impossible to fulfill the requirements here by mere didactic teaching or lecturing. Manipulative instruction is required for each and every pupil, and in order to attain the best results, it ought to be by those having a large experience in both the work and in teaching. It is unfortunate that so often the practical part of the work is committed to the charge of those who have but a limited experience in the work, and a less experience in the communication of their knowledge. Such methods often bring a sad experience to the student.

Not only in the technical but in the didactic teaching is there such a thing as overloading a class-room. In all institutions of learning the methods are constantly undergoing modification, and the tendency is definitely to a closer contact and intimacy between the teacher and the pupil. The old method of continuous and persistent lecturing is giving place to class-room exercises, quizes, recitations, illustrations, and essays. Such exercises are vastly more effective with a limited number of students. The number ought to be such that the teacher could know personally each and every one, and know the peculiarity and disposition of each. Then, and then only, can the pupil be best trained. During the coming year there will doubtless be over three thousand students in the various dental colleges of the country, and the question comes to mind, How many of them are well-fitted for the work upon which he is about to enter? And further, what will be the outcome in respect to their preparation for the duties which they propose to assume?

There is without doubt progress being made in the methods employed in the education of dental students among our colleges, but there is sometimes ground for impatience when methods that are of acknowledged merit and value are available and practicable, and no disposition to employ them is manifested.

It is to be hoped that more care will be exercised in the admission of students this year than ever before, and that those who go out with the endorsement of our colleges hereafter shall possess such attainments and preparation as shall meet the approval of all interested.

